# Enhancing the utility of Proteomics Signature Profiling (PSP) with Pathway Derived Subnets (PDSs), performance analysis and specialised ontologies

**DOI:** 10.1186/1471-2164-14-35

**Published:** 2013-01-16

**Authors:** Wilson Wen Bin Goh, Mengyuan Fan, Hong Sang Low, Marek Sergot, Limsoon Wong

**Affiliations:** 1 Department of Computing, Imperial College London, London, United Kingdom; 2 Department of Computer Science, National University of Singapore, Singapore, Singapore; 3 NUS Graduate School for Integrative Sciences and Engineering, Singapore, Singapore; 4 National Research Foundation, Singapore, Singapore; 5 Department of Pathology, National University of Singapore, Singapore, Singapore

## Abstract

**Background:**

Proteomics Signature Profiling (PSP) is a novel hit-rate based method that proved useful in resolving consistency and coverage issues in proteomics. As a follow-up study, several points need to be addressed: 1/ PSP’s generalisability to pathways, 2/ understanding the biological interplay between significant complexes and pathway subnets co-located on the same pathways on our liver cancer dataset, 3/ understanding PSP’s false positive rate and 4/ demonstrating that PSP works on other suitable proteomics datasets as well as expanding PSP’s analytical resolution via the use of specialised ontologies.

**Results:**

1/ PSP performs well with Pathway-Derived Subnets (PDSs). Comparing the performance of PDSs derived from various pathway databases, we find that an integrative approach is best for optimising analytical resolution. Feature selection also confirms that significant PDSs are closely connected to the cancer phenotype.

2/ In liver cancer, correlation studies of significant PSP complexes and PDSs co-localised on the same pathways revealed an interesting relationship between the purine metabolism pathway and two other complexes involved in DNA repair. Our work suggests progression to poor stage requires additional mutations that disrupt DNA repair enzymes.

3/ False positive analysis reveals that PSP, applied on both complexes and PDSs, is powerful and precise.

4/ Via an expert-curated lipid ontology, we uncovered several interesting lipid-associated complexes that could be associated with cancer progression. Of particular interest is the HMGB1-HMGB2-HSC70-ERP60-GAPDH complex which is also involved in DNA repair. We also demonstrated generalisability of PSP using a non-small-cell lung carcinoma data set.

**Conclusions:**

PSP is a powerful and precise technique, capable of identifying biologically coherent features. It works with biological complexes, network-predicted clusters as well as PDSs. Here, an instance of the interplay between significant PDSs and complexes, possibly significantly involved in liver cancer progression but not well understood as yet, is demonstrated. Also demonstrated is the enhancement of PSP’s analytical resolution using specialised ontologies.

## Background

Proteomics Signature Profiling (PSP) is a novel method for overcoming small sample size, consistency and coverage issues in proteomics [1]. The underlying idea for this method is, despite the lack of consistency in reported proteins, detection is nonetheless context-dependent (the said context being components of conserved and implicated biological complexes or biological pathways) and therefore could be informative if exploited appropriately.

This idea was tested in a published study of Hepatocellular Carcinoma (HCC) patients (5 in moderate and 7 in poor stage) [2]. Utilising real and predicted protein complexes, it is possible to strongly recover patient subclasses in agreement with histopathology. Feature selection also identified liver cancer-associated complexes involved in apoptosis, cell cycle regulation, etc.

It is remarkable this is achievable in spite of little inter-patient agreement. To highlight this, in mod-stage patients, only 25 out of over 800 proteins are common to all 5 patients. Of these 25, all are also reported in poor-stage patients with relatively high counts (≥ 4 out of 7 patients) with the exception of HSP90AA2 and TRAP1 (≤ 2 out of 7 patients). In poor stage, only 3 out of over 1000 proteins are common to all 7. Of these, 2 (CLU and CSTB) are reported in mod-stage patients. LYZ or lysozyme, is the only detected protein common to all 7 poor patients and not found in mod-stage patients. The large disparities between reported proteins meant that it was difficult to select potential biomarkers, and also not possible to generate meaningful hierarchical clustering based on detected proteins. Additional file 1: Figure S1 highlights the extent of poor analytical resolution if hierarchical clustering is performed on the data as it is; the underlying patient classes cannot be recovered.

As a follow-up study, we identified four essential points to address: Firstly, we show that pathways can also be used with the PSP approach. While complexes provided a biologically-rich feature set, it is limited. Pathways are also suitable networks on which PSP could be utilised but has several caveats. 1/ Size and topology: Individual pathways vary considerably in size. Suppose that only a small portion of a large pathway is involved, standard statistical tests would fail. 2/ Coverage and consistency issues: Current pathway databases have abysmal agreement even on the most well-studied pathways. We recently developed an integrated pathway database, PathwayAPI [3], which utilises a novel pathway merging approach based on pathway name matches (Longest Common Substring matching; LCS) [3]. However, the value of this integration has never been demonstrated in a functional study. Furthermore, it is not known how using individual pathway databases---e.g., KEGG or IPA---would affect or skew analysis.

Secondly, as pathways are large biological entities with specialised processes, significant PSP-derived complexes and PDSs that co-locate on the same biological pathways, might engage in novel interplays that may account for cancer progression. This is worth examining further.

Thirdly, the actual false positive rate needs to be better understood, as it was not studied in the original paper. As PSP uses all detected proteins, the possibility of high false positive rates could be a concern. While we demonstrated theoretically in the PSP paper why false positives are negated, we did not show in actual terms its severity or negligibility.

The fourth aim of this study is to demonstrate PSP’s generalisability and to enhance its analytical resolution using specialised ontologies. On the latter, many functional studies are reliant on, but also limited by, the analytical resolution of Gene Ontology (GO). GO was developed for general purposes and, on its own, might not yield specialised/advanced insights especially when a specific biological context---e.g., cancer---is being considered. Here, we use a lipid ontology based on GO for uncovering novel lipid-associated complexes implicated in liver cancer progression. On generalisability, we demonstrate that PSP also works in a non-small-cell lung carcinoma (NSCLC) dataset comparing two subtypes, adenocarcinoma (ADC) and squamous cell carcinoma (SCC).

## Results and discussions

### Significant PDSs are involved with cancer-associated functionalities

A PDS is a pathway-derived and biologically coherent feature determined by the proteomics data. It is a connected subnet within a biological pathway, which is more likely to function together based on the expression data (See methods for details). There are a number of prior works on extracting subnetworks from (large) interaction databases, e.g., Ideker *et al.*[4] and Rajagopalan and Agarwal [5]. While it may also be possible to adapt these other subnetwork methods for extracting PDSs, we feel it is probably best left to a separate (preferably, independently conducted) comparative study. Here, we focus instead on a proof-of-concept to demonstrate that subnets extracted from pathways also work with the PSP approach.

From PathwayAPI, 87 PDSs are derived from the non-merged pathways (derived from 75 different pathways; Additional file 2: Table S1), of which, 23 belong to pathways merge-able in PathwayAPI via LCS. The PDSs are of reasonable sizes, about 70% are size 10 and above (Additional file 3: Figure S2). There does not appear to be a bias for any particular pathway, with most pathways contributing just one and in some rarer cases, two PDSs; see Additional file 2: Table S1.

**Figure 1:**
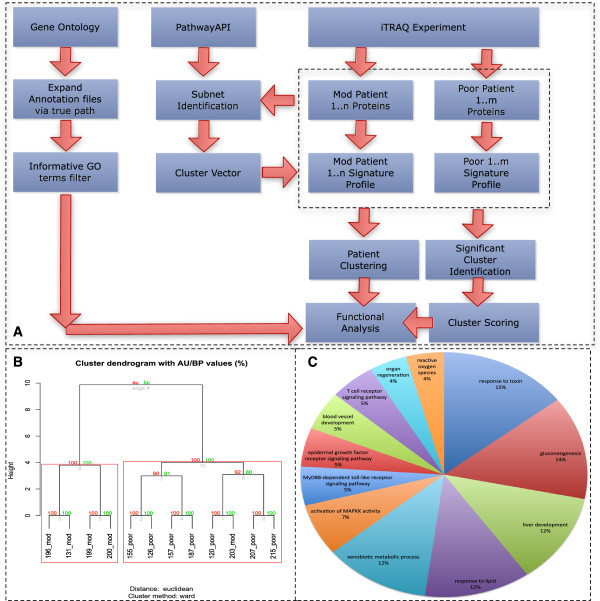
**Analytical pipeline, clustering results and GO term distributions. A:** Detected proteins in mod- and poor-stage HCC patients are used to build PDSs (Pathway-Derived Subnets) from an integrated pathway database (PathwayAPI). These PDSs are used for calculating hit rates for each patient to generate a PSP. The set of PSPs are used for sample class analysis as well as significant feature identification. **B:** Sample class analysis PDSs have sufficient resolution to segregate mod- and poor-stage patients with high confidence.** C: **Significant GO term distribution A large number of significant GO terms are associated with metabolic functions. Although cancer-associated terms such as apoptosis, growth and immune responses are also uncovered. This is consistent with earlier observations based on this dataset

To observe any shared functional themes, significant GO terms for each of these PDSs are identified. We are also interested in the proportional representation of the significant GO terms across all significant PDSs (Additional file 4: Table S3). Unsurprisingly, many of the PDSs are associated with metabolism; Figure 1C. However, we also observed a good number of significant PDSs involved in deregulated metabolism, unstable DNA, cellular proliferation and self-sufficiency in growth signals, inflammation and immunity, angiogenesis, metastasis and invasiveness, and avoiding cell death. To emphasize how the terms are related to cancer, the significant GO terms are further arranged in terms of the major cancer hallmarks [6]; see Additional file 5: Table S4.

### Effects of pathway merging on PSP/PDS performance

While Soh *et al.*[3] showed that pathway merging via string name matching (LCS) is a more robust approach than based on gene and gene interaction overlaps, the effect of this merging method is not known in any sort of functional analysis. We would like to understand how this integration affects analysis.

Extracting subnets from merged PathwayAPI data returned 82 PDSs. The small drop of 5 PDSs (from 87) suggests that merging the pathways (based on the LCS approach) did not make any major change to the overall results. But since slightly less PDSs are produced for merged pathways, they must have been merged into the other PDSs. Suppose PDSx (among the 5 absorbed PDSs) got merged with PDSy (among the remaining PDSs) in some merged pathway P. This must mean that PDSx and PDSy hit two different parts of P that were not contiguous in the un-merged source pathways Px and Py of P. But Px and Py got connected in P after merging.

Clustering of the 87 PDSs from non-merged PathwayAPI did reveal they are also capable of segregating the patients into their respective subclasses with high confidence (AU score = 100). The red and green numbers refer to the AU (Approximately Unbiased) and BP (Bootstrap Probability) p-values (between 0 and 100) respectively. Higher values denote higher confidence. Red squares indicate largest possible clusters where the AU p-value is above 95. Additionally, mod patient 203, which was previously found to be anomalous, was once again found in the poor-stage cluster (Figure 1B).

Clustering the 82 PDSs from merged PathwayAPI recovered the same tree topology. However, the AU score dipped slightly to 99. While the PDSs and significant biological complexes from PSP are vastly different, the topology of the clustering tree is essentially similar, with strong segregation of both mod and poor class patients; Additional file 6: Figure S3.

These results suggest that the LCS merging procedure in PathwayAPI does maintain the integrity of pathway information. But beyond this automated approach, more needs be done, e.g., manual/expert curation to ensure functional congruity and data quality. After all, pathways are very intricately joined, and contain many common members/edges (which also causes the problem with data integration in pathway databases). To illustrate this point, a network was built based on taking a naïve union of all existing pathways in PathwayAPI. There were no isolated components in this highly inter-connected system making it difficult to disambiguate the various constituent pathways. More importantly, attempting to extract PDSs from this results in one super component of size 350, and 3 components of sizes 4, 4 and 6. This lack of informative network features confounds the PSP profiling approach.

### Comparative analysis between PathwayAPI and its constituent databases

PathwayAPI (both merged based on the LCS approach and non-merged) outperforms its constituent databases, demonstrating the value of integration (Additional file 6: Figure S3). Of the constituent databases, KEGG performs slightly worse than PathwayAPI but the topology of the tree is similar. Wiki performs second best: it keeps the general order of the patients but does not have sufficient information to reach significance in separating the patient classes (threshold AU score ≥ 95). IPA is generally similar to Wiki as well, but the mod 203 and poor 120 branch is translocated to the mod group instead.

In any case, the two trees (IPA and Wiki) are of lower confidence than KEGG’s, as well as those based on PathwayAPI and real/predicted complexes. The reason for this is due to the number of features (PDSs and significant PDSs) contributed by each database. Altogether, 58 out of 87 PDSs reached significance at p ≤ 5%, indicating they are strongly discriminative between mod and poor stages; see Additional file 7: Table S2. The high proportion of significant PDSs is because they are already pre-pruned to just the pathway regions where variation between mod and poor is expected to occur.

On the individual pathway databases, KEGG contributes 43 significant PDSs out of 65 PDSs derived from 172 pathways in KEGG, Wiki 6 significant PDSs out of 11 (82 pathways), and IPA 9 significant PDSs out of 11 (45 pathways) respectively (to make a total of 58 significant out of 87 total PDSs (299 pathways) in PathwayAPI. Due to the smaller number of resolving features (PDSs), it is not surprising that Wiki and IPA perform poorer in their ability to resolve the patient samples properly.

Given this result, we cannot over generalise on the respective quality of the individual databases. But the key implication here is that one should never defer to the results from one database solely. Obviously, coverage discrepancies between different databases can result in variations of analytical outcomes. It is thus best to refer to an integrated data resource.

### Significant PDSs and complexes are enriched for co-location on pathways

We use the term “involved” to suggest that a pathway is implicated in HCC if it gave rise to a signficant PDS.

There are a total of 159 significant PSP complexes (i.e., biological complexes that are significantly differential between mod and poor stage). The significant PDSs originate from 58 different “involved” pathways. The number of non-involved pathways is therefore 241 (299–58). On the other hand, 20% (12 / 58) of the involved pathways overlap with a significant complex while 11% (27 / 241) of the non-involved pathways overlap with a significant complex. Hence, an involved pathway is ~ 2x (20/11) more likely than a non-involved pathway to overlap a significant complex; Additional file 8: Table S5.

Also, none of the 364 (523–159) non-significant complexes overlap with any of the 75 involved pathways, while 10 non-significant complexes overlap some of the 241 non-involved pathways. The propensity for significant complexes to co-localise onto the same pathway with a significant PDS is therefore notably high.

To show that the co-localisation effect is not due to the effect of the same proteins being found on both the PSP complexes and PDSs, we looked at the distribution of overlaps between PDS and PSP complexes co-located on the same pathway. In 70% of cases, the Jaccard Score (i.e., intersection over union) is much smaller than 0.2, indicating that this was not due to the effect of the shared proteins.

### A novel molecular switch implicated in HCC progression

**Figure 2:**
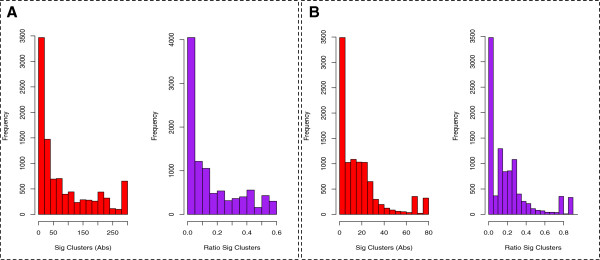
**Left graph (red) shows absolute count distribution of false positive features while right (purple) is the distribution of proportion (false positive features/total number of features).** At 5% significance, the left shift of peaks is within expectation. However, the frequency distributions is still rather high, implying internal clustering among poor patients. This is expected given high variability of reported proteins between poor stage liver cancer patients

The co-locating PDSs and PSP complexes (onto the same pathway) should function in a coordinate manner (i.e., their corresponding cluster scores/expression values should correlate); and generally, this is true. The cluster score correlations between co-located PDS and PSP complexes were examined for both mod and poor stage; Figure 2. Although the cluster scores are clearly correlated, this effect is more pronounced in the poor stage (P = 0.0014, R-square = 0.47). In the mod stage, the correlation is skewed by the presence of two outliers which are relatively high scoring in PDS but low for complex. Removing the two outliers improved the p-value to 1.5e-05 from 0.0077 and the regression fit (R-square) to 0.76 from 0.34. In poor stage, both corresponding PDS and complexes are consistently high.

We examined these two outliers, and found that they corresponded to two different non-overlapping complexes that co-locate on the same pathway (“Purine metabolism”). In the mod stage, the scores for these two complexes are low while the PDS is high. In the poor stage, this disparity is negated: It is high in both PDS and complex. It is possible that this is a switch mechanism that may be involved in the progression from mod to poor stage.

The major site of purine synthesis is in the liver. Interestingly, it is known early on that enzymes involved in purine metabolism play a role in cancer. In fact, an enzymic imbalance, specific only to liver cancer, was known as early as 1983 [7]. However, the mechanistic details are only recently being uncovered. Pang *et al*. [8] recently showed that defects in purine metabolism leads to substantial incorporation of xanthine and hypoxanthine into DNA. This in turn induces mutations, a key driver for oncogenic progression.

We decide to investigate further by analysing the functions of the co-localised complexes: “DNA synthesome complex” and “TNF-alpha/NF-kappa B signaling complex 5”. The former is involved in fat signaling (GO:0048015 phosphatidylinositol-mediated signaling, P = 0.003), cellular immortality (GO:0010833 telomere maintenance via telomere lengthening, P = 4.9e-32), DNA repair (GO:0000724 double- strand break repair via homologous recombination, P = 0.048; GO:0006284 base-excision repair, P = 3.4e-10; GO:0006283 transcription-coupled nucleotide-excision repair, P = 5.0e-19) and cellular proliferation (GO:0000082 G1/S transition of mitotic cell cycle, P = 1.7e-12). The latter is involved in stress/immune responses (GO:0045087 innate immune response, P = 1.7e-07; GO:0043123 positive regulation of I-kappaB kinase/NF-kappaB cascade, P = 2.4e-06), DNA repair (GO:0006283 transcription-coupled nucleotide-excision repair, P = 0.002), cell death (GO:0006916 anti-apoptosis, P = 0.005) and cellular proliferation (GO:0000082 G1/S transition of mitotic cell cycle, P = 0.02; GO:0016032 viral reproduction, P = 0.01). It is clear that both complexes are involved in cancer-associated functions, in particular, DNA repair.

The associated PDS appears to work primarily on metabolic and purine-related functions, including GO:0009161 ribonucleoside monophosphate metabolic process (P = 0.0001) and GO:0006144 purine base metabolic process (P = 7.9e-09). However, it is also related to differentiation (GO:0002761 regulation of myeloid leukocyte differentiation, P = 0.03) although myeloid differentiation is typically associated with leukemias, not with liver cancer.

Since the expression level of the PDS is both high in mod and poor stage, it is possible that this is an early requirement for oncogenic transformation. Indeed, upsetting nitrogenous base balances can lead to increased chance of error in DNA replications. Our identified PDS suggests that this is the region of the purine metabolism pathway most differentially affected. But in order for oncogenic progression to occur or speed up (for transition from mod- to poor-stage liver cancer), it is logical, and indeed as we find, to target protein complexes that are involved in rectifying mistakes in DNA replication or effecting DNA repair. To further implicate these two out of several other candidate DNA repair complexes, they are closely associated with the pathway as well. In light of these results, the biological relationship between the purine metabolism PDS and the associated PSP complexes should be further explored experimentally.

### PSP is a powerful and precise method with reasonably low false positive rates

There are 523 biological complexes found in CORUM (size ≥4) used in PSP. At p ≤ 5%, approximately 523 * 5% ≈ 27 false positives are expected. Similarly, there are 87 PDSs, and thus at p ≤ 5%, approximately 87 * 5% ≈ 5 false positives are expected. In order to check the empirical false positive rates against these theoretical estimates, we used the 7 poor-stage patients, randomly assigned them into two groups, and applied PSP analysis. Since both groups were actually poor-stage patients, all significant complexes / PDSs resulting from this analysis should be considered false positives.

**Figure 3:**
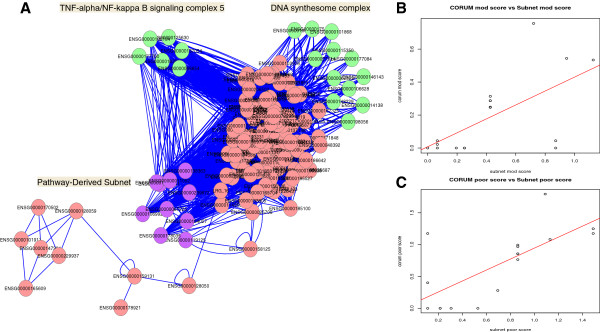
**False positive distribution for PSP (A) and PDS (B).** Co-localisation and expression profile of PDSs and complexes (DNA synthesome and TNF-alpha/NF-kappa B signaling complex 5) on the purine metabolism pathway. **A**: The purine metabolic pathway is shown as an undirected graph. Significant PSP clusters are highlighted in green while significant PDS is shown in purple. **B** and **C**: Regression plots for expression scores of protein complexes and PDSs co-located on the same biological pathway in mod and poor stage respectively. Enveloped in a checked circle are two complex outliers that are low expressing in the mod stage relative to the co-locating PDS but swung to overexpression in the poor stage

Figure 3 shows the distribution of false positives across 10,000 randomisations for the poor-stage patients. In each round, the set of significant complexes or PDSs (false positives) are reported at p ≤ 5% (with Benjamini-Hochberg correction). Figure 3A and 3B shows the false positives reported for CORUM (mean ≈ 80, median = 40, mode = 6) and PathwayAPI (mean ≈ 18, median = 12, mode = 3) respectively. The red bar shows the absolute false positive counts while the purple bars are normalised to 1. The first two peaks in Figure 3A and 3B are within expectation and indicate a low false positive rate. However, the overall distribution of peaks, as well as the mean number of significant clusters over 10,000 randomisations are slightly higher than expected. This is likely due to the presence of internal clusters among the poor-stage patients as well as small sample size; Additional file 1: Figure S1.

While the false positive analysis may be more ideally executed by using technical repeats from say, a cell line. We nonetheless show here that these hit-rate based methods utilising biological pathways and complexes are powerful and precise.

### Identification of novel lipid-associated complexes involved in cancer progression

The liver is a major metabolic center, and a primary regulation site of lipids. Current evidence suggest that lipids are not mere energy-providing metabolites but can effect profound changes via various signaling pathways. This is not limited to cholesterol-based signaling molecules, which are more well known. There are over 2,000 species of lipids, most of which have poor characterisation and not easily analysable [9]. Lipid dysregulation is also commonly associated with liver cancer [10].

Many proteins are annotated to specific functionalities based on GO annotation rules but the lipid associations are not well known or sufficiently comprehensive.

To overcome this and to maximise compatibility with current annotation standards, we developed a set of gold-standard lipid-related terms based on GO terms; see Methods. This list consists of 1463 lipid-associated GO terms in the Biological Processing category (BP terms), 924 GO terms in the Cellular Component category (CC terms) and 1736 GO terms in the Molecular Function category (MF terms).

For BP, out of a total of 177 PSP predicted/real complexes, 21 reached significance. Of these 21, 5 are predicted clusters. Therefore, about 12% of significant PSP complexes are lipid associated. About half of these are not previously known to be lipid associated . A list of these novel/potentially novel BP lipid-associated real complexes are reported in Table 1, and a full list (with further details) is given in Additional file 9: Table S6.

**Table 1:** List of potentially novel and novel lipid associated complexes implicated in liver cancer

**Novelty**	**CORUM ID**	**Complex name**	**Function**	**Lipid involvement**
PN	1096	SNX and PDGF receptor complex	reported to be involved in transport and transmembrane signal transduction	lipid involvement yes
PN	1104	Transferrin receptor complex	reported involved in transport and receptor mediated endocytosis	lipid association yes
PN	563	Complex V; F1F0 ATPase	energy production, mitochondrial processes, particularly in heart muscle	lipid involvement yes
PN	654	BLOC1-BLOC2 complex	transport and targeting	lipid involvement potentially novel
PN	142	CD147-gamma-secretase complex (APH-1a, PS-1, PEN-2, NCT variant)	signaling, protein fate	lipid involvement yes
PN	652	AP3-Bloc1-complex	transport	lipid involvement yes
PN	657	Retromer complex (SNX1, SNX2, VPS35, VPS29, VPS26A)	protein targeting and transport	lipid involvement yes
PN	2837	Profilin 1 complex	cytoskeleton organisation, endocytosis, potential metastasis involvement	lipid involvement yes
PN	1060	Retromer complex (SNX1, SNX2, VPS35, VPS29, VPS26B)	transport	lipid association yes
N	280	HMGB1-HMGB2-HSC70-ERP60-GAPDH complex	DNA repair, nucleic acid binding, response to stress and DNA damage stimulus);high cancer association	lipid involvement not immediately decipherable
N	312	Cell cycle kinase complex CDK4l	cell cycle control, cancer association yes	lipid involvement not immediately decipherable
N	247	RalBP1-CCNB1-AP2A-NUMB-EPN1 complex	cell cycle control, endocytosis	lipid involvement not immediately decipherable
N	5230	CHUK-NFKB2-REL-IKBKG-SPAG9-NFKB1-NFKBIE-COPB2-TNIP1-NFKBIA-RELA-TNIP2 complex	Signaling, cancer association yes	lipid involvement not immediately decipherable
N	311	Cell cycle kinase complex CDK2	cell cycle control, cancer association yes	lipid involvement not immediately decipherable
N	5423	HSP70-BAG5-PARK2 complex	protein folding and stabilisation	lipid involvement not immediately decipherable
N	2390	CD98-LAT2-ITGB1 complex	cell adhesion, may define cell polarity which in turn has to do with invasiveness	lipid involvement not immediately decipherable

*PN – potentially novel, N – novel.

Among the novel lipid-associated complexes, the HMGB1-HMGB2-HSC70-ERP60-GAPDH complex is of particular interest. This complex was isolated from human leukemia cells deficient in components of the mismatch repair system (Nalm6) and functions by detecting changes in DNA structure caused by incorporation of nonnatural nucleosides and is a determinant of cell sensitivity to DNA modifying chemotherapy [11]. Disruption of DNA-repair mechanisms is important in driving liver cancer progression from early to late stage [12]. Moreover, this cluster exhibited a 2.5 fold increase in expressional level. Its lipid involvement appears to be dispersed however: It is involved in both golgi-mediated transport and phosphoinositide-mediated signaling. Also interesting are that the HMG family of proteins are associated with malignant neoplasias [13], in particular, HMGB1 was identified as a potential cancer therapeutic target [14].

**Figure 4:**
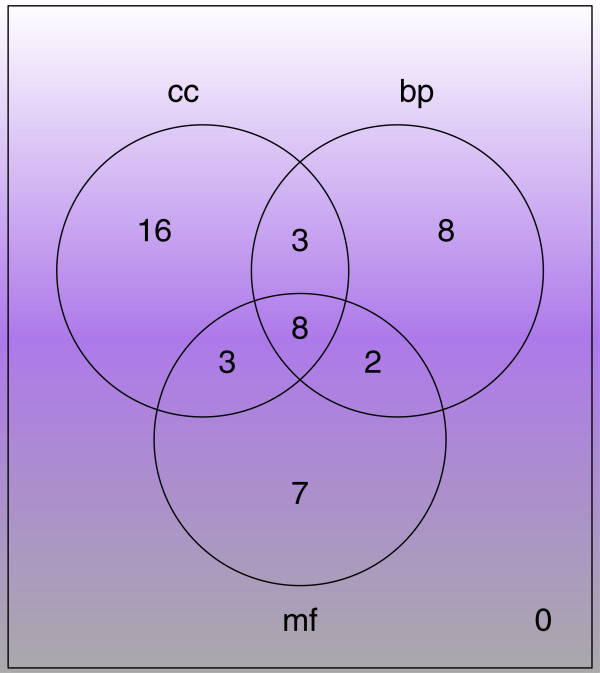
**Overlaps between significant complexes identified via lipid-associated GO BP, CC and MF terms.** The overlaps between significant lipid-associated clusters identified via lipid-associated GO BP, CC and MF terms are expressed as a venn diagram. For all 3 categories, about at least half of the complexes are shared between two categories

To understand if the other lipid GO categories also report similar results, a similar analysis was repeated for CC and MF terms, reporting 30 and 20 significant complexes respectively.For the full list of complexes, refer to Additional file 10: Table S7. Figure 4 shows the overlaps between the significant lipid-associated complexes derived from each GO category. For MF and BP, more than half of the complexes are shared with the other categories. For CC, this is slightly less than half. 8 significant complexes are common to all three categories. These include Retromer complex (comprising SNX1, SNX2, VPS35, VPS29, VPS26B; involved in transport), SNX complex (comprising SNX1,1a,2,4, PDGF receptor; transport and signaling), SNX complex (comprising SNX1a, SNX2, SNX4, TFRC; transport), RalBP1-CCNB1-AP2A-NUMB-EPN1 complex (cell cycle), and Retromer complex (comprising NX1, SNX2, VPS35, VPS29, VPS26A; transport). The remaining 3 clusters are predicted.

Although BP is commonly used for functional annotation and analysis, the limited overlaps between the 3 categories suggest that it is prudent to examine all three GO categories when determining lipid associations.

### Generalisability using non-small-cell lung carcinoma samples

To demonstrate generalisability, we performed similar analysis using the results from Wei *et al.*[15]. Here, two subtypes of non-small-cell lung carcinoma (NSCLC), adenocarcinoma (ADC) and squamous carcinoma (SCC) were analyzed. We used the results from 2-dimensional LC-MS/MS with multiplexed selective reaction monitoring (SRM, or MRM). This dataset has less consistency issues compared to the HCC dataset. As such, the authors were able to analyse it using standard hierarchical clustering.

However, there are variabilities, though limited, in terms of identified proteins for each sample (n = 30). On average, there are about 3000 identified proteins in total, which is 3 times more than the HCC dataset.

**Figure 5:**
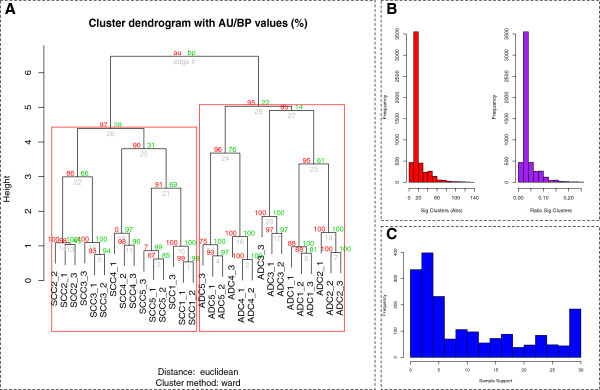
**Generalisability tests using NSCLC dataset. A:** The underlying patient subclasses can be recovered with high confidence using complexes. **B:** The left histogram shows the absolute (Abs) count of significant clusters per randomisation. The right histogram is the number of significant clusters normalised by the total number of randomisations (ratio sig clusters). **C: **Protein support over 30 samples. Most proteins are only supported by a subset of samples

Figure 5A shows that the underlying patient subclasses can be recovered with high confidence using complexes. The estimated number of false positives (average = 19.93, median =15, mode =12) for this was also within the expected limit---523 complexes * 5% ≈ 27 false positives are expected; Figure 5B. The left histogram shows the absolute (Abs) count of significant clusters per randomisation. The right histogram is the number of significant clusters normalised by the total number of randomisations (ratio sig clusters).

Figure 5C shows the extent of protein support across all 30 samples analysed. Most proteins are supported by few of the samples. But unlike the original HCC dataset studied in PSP, the extent of variability is much reduced here.

As mentioned earlier, the NSCLC dataset reported 3 times as many proteins than the HCC dataset. Since PSP is dependent on variations in reported proteins per sample, it may not work if the assay reports lots of proteins. However, we find that the effectiveness of PSP is undiminished.

Current proteomics assays also saw the rise of extensive set ups allowing reporting of more than 10,000 proteins in samples [16,17]. However, these datasets are incompatible with the PSP approach as the premise of these are on deep proteome investigation of single cell lines. PSP requires variability between samples, and in a typical proteomics set up, achieving more than 10000 protein identifications is uncommon.

## Conclusions

PSP is a powerful and precise method, with acceptable false positive rates. This is confirmed in the use of both biological complexes as well as PDSs.

The PDSs are informative, being able to properly recover the patient classes as well as selecting significant features whose enriched GO terms are consistent with the liver cancer phenotype.

Pathway analysis based on integrated data is evidently superior due to consistency and coverage issues among databases. KEGG performs well with our data but the added PDSs from WikiPathways and IPA improved analytical resolution.

By analysing the inter-play between co-localised significant PDSs and complexes, we find very good expression correlation implying coordinate responses and activity. We also uncovered an interesting relationship for two DNA repair complexes with the “purine metabolism” pathway. This co-localisation appears to be involved in mod-to-poor-stage liver cancer transition and warrants further biological exploration. Finally, we also demonstrate how analytical resolution can be enhanced via the use of specialised ontologies. Specifically, we have identified several novel lipid-associated complexes from the set of significant PSP complexes.

## Methods

### Data sources

#### Patient sample preparation and proteomics profiling

Briefly, liver tissues were obtained from 12 HCC-diagnosed male patients with chronic Hepatitis B virus (HBV) infection. Tissues collected were grouped according to histology report; 5 had moderately differentiated HCC (mod) and 7 had poorly differentiated HCC (poor). Paired tissues were obtained from each patient, one from the adjacent non-tumor region (normal) and the other from the tumor region of the resected liver. Samples were labelled with iTRAQ-tags and separated using two phase LC-MS.

Proteomics analysis was performed using GPS Explorer Ver. 3.6. Peptides and proteins were determined using Paragon6 (Protein Pilot Ver. 4; AB SCIEX) and MASCOT5 (Ver. 2.1; Matrix Science) on IPI human database (Ver. 3.31). Quantification of iTRAQ ratios and standard deviation (s.d.) was performed using standard parameters on GPS Explorer or Protein Pilot.

In Goh *et al.*[12], it was found that the reported proteins from both databases (MASCOT and Paragon) corresponded well in terms of ratios and ranks. Paragon is more senstive than MASCOT and produced better results in the original PSP paper. Hence in this work, only the Paragon results are used. For details, refer to Additional file 11: Supplementary Methods.

#### PathwayAPI

For the reference pathway database, we utilised Pathway API [3], which comprised data from three major pathway repositories: the Kyoto Encyclopedia of Genes and Genomes (KEGG) [18], WikiPathways [19] and Ingenuity Pathways (IPA) (http://www.ingenuity.com). It consists a total of 299 gene pathways, 21,314 genes and 60,900 gene pairs.

#### Identifying proteins for candidacy in the PDSs

As a whole pathways can be quite large, analytical resolution can be enhanced by considering only the relevant subnets. A PDS is a pathway-derived and biologically coherent subnet. To determine a PDS, detected proteins found in at least half of the patients in mod and poor stage respectively were mapped to each pathway. Expression filters (i.e., the levels of expression of the proteins) were not used as they resulted in a very sparse number of small subnets. Unmapped pathway proteins are removed, causing the pathway to fragment into connected components or subnets. Subnets of minimum size 4 were extracted for analysis as PDSs. The minimum size requirement reduces large fluctuations in hit rates due to small sizes. The derived PDSs are treated as a cluster vector of features.

#### Clustering and feature selection

In the PSP paper [2], each feature C in the cluster vector is a protein complex from the CORUM database. But here PDSs are used instead. Given two sets A and B of proteomic profiles from two phenotypes respectively (mod and poor), for each PDS C, and each patient i in A, the hit rate HCAi, is defined as the overlap between the PDS C and the proteins detected in patient i divided by the PDS size. Similarly, we compute HCBj for each PDS C and patient j in B. The total set of hit rates for each patient across the set of PDSs is the patient’s signature profile.

These profiles are used to examine the consistency and confidence of the derived relationships between samples. For simplicity, hierarchical clustering was used to examine sample relationships. Distances between individual profiles were calculated using Euclidean, while Ward’s was used to determine the clustering.

To evaluate confidence in the clustering results, the R bootstrap resampling package pvclust was used. For the tree generated, p-values (between 0 and 1) are calculated. pvclust provides two types of p-values: AU (Approximately Unbiased) and BP (Bootstrap Probability). AU, which is computed by multiscale bootstrap resampling, is a better approximation to unbiased p-value than BP value which is computed by normal bootstrap resampling.

To perform feature selection, for each PDS C in the cluster vector, two lists are produced *HA* = 〈*HCA*_1_,…, *HCA*_*m*_〉 and *HB* = 〈*HCB*_1_, …, *HCB*_*n*_〉, where A and B correspond to the mod and poor stages. The t-statistic score between the lists HA and HB is then computed by the standard formula:

$$t\_score=\frac{\bar{H}A-\bar{H}B}{S_{HA,HB}\sqrt{\frac{1}{n}+\frac{1}{m}}}$$

where

$$S_{HA,HB}=\sqrt{\frac{\left(m-1\right){S_{\mathrm{HA}}}^{2}+\left(n-1\right){S_{\mathrm{HB}}}^{2}}{m+n-2}}$$

If the t-statistic is significant, then the PDS C is differentially expressed between the mod and poor stages. As the calculated t-score may not necessarily follow a standard t-distribution, weighted randomisation via class label swapping was performed between mod- and poor-stage samples 10,000 times to produce the null distribution. If the t-score value is negative (positive), the empirical p-value is determined by the percentage of null-distribution t-scores that are smaller (greater) than the actual t-statistic value.

For those PDSs regarded as significant (p ≤ 5%), we calculate a ranking score for the mod and poor stage respectively using the reported iTRAQ protein ratios. Suppose we have a PDS comprised of proteins A, B, C, D. A is supported by 4 mod-stage patients with ratio (1.1, 0.8, 1, 1.2), B is supported by 1 patient with ratio of 5 while C and D are not supported. If the ratio is lower than 1, we convert it by taking its reciprocal. To find out how big is this ratio, we take difference = ratio – 1. The score, S, would thus be Σ(0.1, 1/0.8 - 1, 0, 0.2) + 4. However, with this scoring approach, PDSs with more proteins tend to be ranked high. For example, a PDS with 10 proteins (A1, … , A10) and patient i has a high ratio value on Ai and low ratio value on the other 9 proteins; this PDS will get a higher score than a PDS of size 4 with all patients having medium ratio values on all 4 proteins in this PDS. To improve the scoring function for such instances, we propose dividing S by the number of unique proteins that were reported in the patient. For example, suppose a PDS consists of proteins A, B, C, D, there are 2 patients reporting A and their scores are 1.1, 1.2 while 1 patient report B and his score is 5. The cluster score is (0.1 + 0.2 + 4)/2 (note that the denominator is not 4).

#### False positive analysis for PDS and PSP

To determine whether the hit-rate based methods (PSP and PDS) were reporting a large number of false positives, poor-stage patients (being the larger group) were divided into groups A and B randomly 10,000 times. A t-score, and accompanying p-value were calculated as before.

#### Gene ontology filtering and cluster functional annotation

GO provides a controlled vocabulary for assessing cluster function and coherence. The annotation files and GO tree files (ver. 1.2) for Homo sapiens, dated 23 April 2011, were downloaded from http://www. geneontology.org. UniProtKB accessions were mapped to Ensembl Gene IDs via Biomart. To refine analysis, informative biological process terms (term that is annotated to at least 30 proteins and has no child term having more than 30 annotated proteins) were extracted from the GO OBO file [20]. Significance testing was performed using hypergeometric test with Bonferroni correction (p ≤ 5%).

#### Identification of novel lipid-associated complexes implicated in liver cancer

Detailed curation rules for lipid-associated GO terms are provided under Supplementary Methods. Briefly, for MF and CC terms, it is based on indirect lipid-term and functional associations. For BP terms, two principles, criticality and generality are applied. The former is concerned with identification of lipid-related key events, for which no alternative steps are possible, while the latter only holds if the association is generalisable across various tissues or species.

The lipid association of a complex is the hit rate (a/n) where a is the number of proteins (in the complex) annotated to at least one lipid-related GO term, and n is the size of the complex. We calculate the p-value using a Functional Class Scoring (FCS)-like procedure [21] where for each real cluster, we generate random clusters of size n 10,000 times, and calculate a randomised lipid content in a similar manner. The p-value is the number of times a random lipid content for a randomised complex is bigger than the original complex divided by total number of randomisations.

Determination of a complex’s novelty is done manually by searching for the presence of lipid-associated annotations or references in the literature. A harder task however, is identifying which lipids are associated with the protein complex. This is non-trivial as there are over 2,000 species of lipids, and many with unknown functions or poor annotations.

## Competing interests

The authors declare that they have no competing interests.

## Authors’ contributions

WWBG and LW conceived the original ideas for this work. WWBG performed the computational as well as biological analysis, and wrote the manuscript. FM and LHS curated the lipid-related GO terms. MS and LW supervised, contributed to refinements and co-wrote the manuscript. All authors read and approved the final manuscript.

## Supplementary Material

Additional file 1: Figure S1 Left: Histograms showing the lack of consistency in identified proteins between patients in the same cancer group (moderate and late). The hierarchical clustering tree on the right demonstrates the poor resolving power of the proteomics data due to lack of consistency, which justifies the need for novel approaches for tackling this issue. Click here for file

Additional file 2: Table S1 The contributions of each pathway to a PDS. Pathway_ID correspond to the PathwayAPI pathway ID while PDS_contribution indicates the number of PDSs that particular pathway contribute based on the liver cancer proteomics dataset. Click here for file

Additional file 3: Figure S2 Size distribution of pathway subnets or PDSs extracted from non-merged PathwayAPI using liver cancer proteomics data. Most PDSs are relatively small, and range from size 5 to 10. Click here for file

Additional file 4: Table S3 GO term distributions among significant PDSs. Click here for file

Additional file 5: Table S4 Sorted significant GO terms according to the cancer hallmark classifications. Click here for file

Additional file 6: Figure S3 Clustered samples using HCL. Significance is calculated the AU score. Significant clusters are enveloped in red rectangles. The performance of clustering using different Pathway databases are shown. Included also is the original clustering result from PSP using biological (CORUM) and predicted complexes. Click here for file

Additional file 7: Table S2 Set of 58 significant PDSs derived from non-merged PathwayAPI. Shown also are the hit-rate p-values, expression scores (mod and poor), constituent members and annotated GO terms. Click here for file

Additional file 8: Table S5 Co-locating PDS and Complexes on same biological pathways. Pathway_id corresponds to PathwayAPI’s reference id. CORUM_id refers to CORUM databases’s index for biological complex. Also included in this table are the constituent members, and expression scores for PDSs and complexes for both mod and poor stage. Click here for file

Additional file 9: Table S6 Predicted lipid-associated complexes. Cluster_ID is the same as CORUM’s unless it is prefixed by a G, which means it is a predicted cluster. The p-value is from PSP, which indicates whether this cluster is significantly differential between mod and poor groups. The lipid-content p-value denotes whether this cluster is significantly lipid-associated. Mod and poor scores indicates the expressional deregulation of the cluster in each cancer phase. Members indicates the constitution of each cluster. Click here for file

Additional file 10: Table S7 Significant clusters found for each GO category for lipid association. Cluster_ID is the same as CORUM’s unless it is prefixed by a G, which means it is a predicted cluster. Click here for file

Additional file 11 Supplementary_Methods Details for the proteomics experimental procedure. Click here for file
