# Supplementary material for: Enhancing the utility of Proteomics Signature Profiling (PSP) with Pathway Derived Subnets (PDSs), performance analysis and specialised ontologies
Source: BMC Genomics. 2013 Jan 16;14:35. doi: 10.1186/1471-2164-14-35 (PMC3636053; doi:10.1186/1471-2164-14-35)
Supplement: Additional file 1: Figure S1 — Left: Histograms showing the lack of consistency in identified proteins between patients in the same cancer group (moderate and late). The hierarchical clustering tree on the right demonstrates the poor resolving power of the proteomics data due to lack of consistency, which justifies the need for novel approaches for tackling this issue. [file 1471-2164-14-35-S1.docx]

Supplementary Methods

# Experimental Procedure

## Patient sample preparation and proteomics profiling

Briefly, liver tissues were obtained from 12 male patients diagnosed with HCC and suffered from cirrhosis with chronic Hepatitis B virus (HBV) infection. There was no reported metastasis at the point of surgery. Tissues collected were grouped according to histology report; 5 had moderately differentiated HCC (mod) and 7 had poorly differentiated HCC (poor). Paired tissues were obtained from each patient, one from the adjacent non-tumor region (normal) and the other from the tumor region of the resected liver.

## iTRAQ labeling

Protein lysates from were first precipitated using the 2-D Clean-Up kit. The protein pellets were subsequently resuspended in either dissolution buffer (500mM triethylammonium bicarbonate and 0.1% (w/v) SDS) for iTRAQ labelling. iTRAQ labelling and processing of the samples were carried out as described by the protocol with minor modifications and using the reagents provided from Applied Biosystems. 100 µg of protein from each sample was reduced with 50mM of TCEP at 6ºC for 1 hr, and subsequently alkylated with 200mM of methyl methanethiosulfonate (MMTS) for 10 min at room temperature. Each sample was diluted to achieve a final concentration of 0.05% (w/v) SDS prior to trypsinization at 37ºC for 16 hr. Following this, each tryptic digest was labeled for 1 hr with one of the four isobaric amine-reactive tags. The labelling was carried out at random ensuring that 2 pairs of patient tissues were labeled as follows: Channel 114 (non-tumour); Channel 115 (tumour); Channel 116 (non-tumour); and Channel 117 (tumour samples). These four iTRAQ-labeled samples were then pooled and passed through a strong cation exchange cartridge as recommended by the manufacturer (Applied Biosystems). This eluate was further desalted using a Sep-Pak cartridge (Millipore), lypholised and reconstituted in appropriate buffers for 2-D LC

## Two-Dimensional liquid chromatography separation of labeled peptides

iTRAQ-labeled peptide mixtures was further separated using an Ultimate^TM^ dual-gradient LC system (Dionex-LC Packings) with a Probot^TM^ MALDI spotting device. A two-dimensional LC separation was performed as follows: the labeled peptide mixture was first dissolved in 2% (v/v) acetonitrile containing 0.05% (v/v) TFA and injected into a 0.3 × 150 mm strong cation-exchange (SCX) column (FUS-15-CP, Poros 10S; Dionex-LC Packings) for the first dimensional separation. The mobile phase A was 5mM KH2PO4 buffer, pH 3, 5% acetonitrile and mobile phase B 5mM KH2PO4 buffer, pH 3, 5% ACN + 500 mM KCl respectively. The flow rate was 6 µl/min. A total of 9 fractions were obtained using step gradients of mobile phase B: unbound, 0-5, 5-10, 10-15, 15-20, 20-30, 30-40, 40-50, 50-100% of B. The eluting fractions were captured alternatively onto two 0.3 × 1-mm trap column, washed with 0.05% TFA and followed by gradient elution in a 0.2 × 50-mm reverse-phase column (Monolithic PS-DVB; Dionex-LC Packings). The mobile phase used for this second-dimensional separation was 2% ACN with 0.05% TFA (A) and 80% acetonitrile with 0.04% TFA (B). The gradient elution step was 0-60% B in 15 min at a flow rate of 2.7 µl/min. The LC fractions were mixed directly with MALDI matrix solution (7mg/ml CHCA and 130 µg/ml ammonium citrate in 75% acetonitrile) at a flow rate of 5.4 µl/min via a 25-nl mixing tee (Upchurch Scientific) before they were spotted onto a 192-well stainless steel MALDI target plate (Applied Biosystems) using a Probot Micro Precision Fraction collector (Dionex-LC Packings), at a speed of 5 sec per well. 50 fmol of ACTH (18-39) peptide (m/z = 2465.199) was spiked into each well as internal standard.

## Mass spectrometry analysis and database search

We analyzed samples previously using a 4700 Proteomics Analyzer mass spectrometer (AB SCIEX) with MALDI source and TOF/TOF optics [^1^](#_ENREF_1)^,^ [^2^](#_ENREF_2). Briefly, MS/MS analyses were performed using nitrogen at collision energy of 1 kV and a collision gas pressure of 1 × 10^-6^ Torr. The GPS Explorer^TM^ software Ver. 3.6 (AB SCIEX) was used to create and search files with the MASCOT[^3^](#_ENREF_3) (version 2.1; Matrix Science) and Paragon[^4^](#_ENREF_4) (Protein Pilot^TM^ version 4; AB SCIEX) search engines for peptide and protein identifications. The International Protein Index (IPI) human database (version 3.31) was used for the search and this was restricted to tryptic peptides. One thousand shots were accumulated for each MS spectrum. For MS/MS, 6,000 shots were combined for each precursor ion with signal-to-noise (S/N) ratio greater or equal to 100. For precursors with S/N ratio between 50 and 100, 10,000 shots were acquired. The resolution used to select the parent ion was 200. No smoothing was applied before peak detection for both MS and MS/MS, and the peaks were deisotoped. For MS/MS, only the peaks from 60 Da to 20 Da below each precursor mass, and with S/N greater than or equal to 10 were selected. Peak density was limited to 30 peaks per 200 Da, and the maximum number of peaks was set to 125. Cysteine methanethiolation, N-terminal iTRAQ labelling, and iTRAQ labeled-lysine were selected as fixed modifications while methionine oxidation was considered as a variable modification. One missed cleavage was allowed. Precursor error tolerance was set to 100 ppm while MS/MS fragment error tolerance was set to 0.4 Da. Maximum peptide rank was set to 2.

The average iTRAQ ratio and standard deviation (S.D.) were determined using the GPS Explorer^TM^ software (version 3.6) or Protein Pilot^TM^ (version 4). The ratio is taken as the tumour sample against adjacent non-tumor region. For MS/MS, only the peaks from 50 Da to 20 Da below each precursor mass, and the minimum S/N filter was designated at 10. The mass exclusion tolerance was 3 Da around 115.5 m/z. Peak density was limited to 50 peaks per 200 Da, and the maximum number of peaks was set to 80.

## Peptide Identification and iTRAQ Quantification

Using GPS Explorer^TM^ from Mascot, the significance threshold was calculated by -10In(*P*) where *P* represents the probability of whether an observed match between an experimental sequence and that from a database is a random event [^5^](#_ENREF_5). A real match will have a low probability of being a random event.

A randomized database was then generated using IPI human database (version 3.31) and used to calculate the false-positive rate (FDR), i.e. , the probability of matching a spectrum to a peptide by chance. The randomized database has the same number of amino acids and candidate peptides as the original IPI database but is composed entirely of artificial peptide sequences. Identifications were obtained by comparing the peptide hits obtained from the random and target databases at different ion score confidence interval (C.I. %) and the FDR calculated. The minimum ion score C.I. % was determined to ensure that no more than 5% FDR was achieved, therefore limiting the expected proportion of incorrect target peptide matches. Since this proportion is not known, FDR is estimated as the number of matches to decoy peptides with the same or better ion score, divided by the total number of matches to target and decoy databases with the same or better ion score C.I. %. Hence proteins are identified from the target database when they are matched to at least two peptides which passed the ion score C.I.% threshold. For single-peptide matched proteins, only those with ion score C.I. % greater than the highest ion score C.I. % obtained from the random database search were selected as significant. In Protein Pilot autobias correction was applied and the Unused ProtScore was > 1.3 (C.I. % > 95%).

iTRAQ ratios were calculated based on the areas of the iTRAQ reporter fragment peaks (114, 115, 116 and 117; Supplementary Figure 5), and the ratios calculation included only peptides identified with confidence interval (C.I. %) above cutoff thresholds. For example, to calculate the average protein fold change of CPS1 of patient #131 (Supplementary Table 1, tab A), the summed signal intensities of peptides tagged with 115 (which represents the tumour sample) were divided with the summed signal intensities of same peptides tagged with 114 (which represents the non-tumour counterpart from the same patient).

In Goh *et al*, it was found that the reported proteins for both databases (Mascot and Paragon) corresponded well in terms of ratios and ranks[^6^](#_ENREF_6). Most Mascot hits were also found in Paragon. In addition, Paragon consistently reported more proteins although we found that these were significantly lower ranked. In both algorithms, the FDR was fixed at 5%. Given that PSP relies on the hit rates of patient proteins against a vector of complexes, the additional proteins may (and indeed as we report in this paper) improve the performance of analysis.

# Controlling for false positives

Suppose the chance of an individual protein being false positive in any patient (regardless of phenotype) is r% (i.e., there is r% noise). Suppose a complex has n proteins. And suppose the real hit rate (i.e., not due to false positive) is h%. Then the observed hit rate on this complex should be ohr = n*h% + n*r% *(1−h%). The (1−h%) is to account for the double counting of proteins that are real hits as false positive. Substituting this into the usual t-score formula, we see that the t-score of a complex in the absence of noise is t_nonoise_ = (n*h_A_% − n*h_B_%)/Δ, while the t-score of a complex in the presence of noise is t_noise_ = ((n*h_A_% + n*r%*(1−h_A_%)−(n*h_B_% + n*r%*(1−h_B_%)))/Δ, where h_A_ (h_B_) is the mean actual hit rate of phenotype A (B) samples on the complex and is the usual square root of the sum of variance. So the difference between t_nonoise_ and t_noise_ is (t_nonoise_ − t_noise_)Δ = n* (h_A_% - h_B_%)*r%. Thus, the greater the noise r% is, the greater the difference between t_nonoise_ and t_noise_. In particular, the t-score with noise (t_noise_) gets smaller as the noise r% increases.

The null distribution t-scores, on the other hand, does not change much because h_Anull_% and h_Bnull_%---the respect hit rates on the complex by the null A- and B-phenotype samples---are expected to be the same. This is because the sample labels are randomized, so the mean hit rate on the complex by phenotypes A_null_ and B_null_ should be the same. This means t_noise_ is shifted towards 0 on the null distribution, while t_nonoise_ remains at the extreme end. Consequently, there is a loss in sensitivity as the noise r% grows. Interestingly, this implies there is a smaller number of false positives as the noise r% grows! For the hierarchical clustering, the distance is also reduced by n* (h_A_% - h_B_%)*r% at each complex. So, as r% increases (i.e., more noise), the distance is reduced more. This causes a loss of resolution in the hierarchical clustering.

## Identification of novel lipid-associated complexes implicated in liver cancer

Lipid-associated GO terms were derived via expert curation following a set of rules. The curation rules for CC and MF terms are as follow: (i) If a term whose name or definition contains explicit keywords relating to lipid, the term is lipid related. (ii) If a term is in the form of “X transporter activity”, we consider the term as lipid related if X is involved in a lipid-related process. (iii) In the case of catalytic activity or reaction, if any substrate or product is lipid related, the term is lipid related. (iv) Polyketides are NOT considered as lipids while lipopolysacharides, glycolipids and lipoproteins are considered as lipid related. (v) Transmembrane signaling receptors are considered lipid-related terms with the explicit mentioning of the keyword “transmembrane”. The curation rules for BP terms are as follow: (i) The BP term’s name or definition contain explicit keywords relating to lipid, which include lipopolysacharides, glycolipids and lipoproteins. (ii) The BP term is in the form of “X metabolic process” and, X is tightly connected to lipid pathways in the sense that: (1) the metabolic process is immediate precursor to a key lipid pathway/process and (2) a majority of X ends up in lipid-related pathways. (iii) The BP term refers to a biological process that contains a key event which involves membrane folding and vesicle formation/fusion. (iv) The BP term refers to a biological process that has a key event which involves modification, breakdown and other catalysis of lipid molecules and complex. (v) The BP terms can be linked to lipids in some other ways in accordance to the following two general principles.

The first principle is criticality: A biological process is series of events accomplished by one or more ordered assemblies of molecular functions. Key events are defined as those that are always present and not replaceable (no alternative steps). BP terms containing lipid-related key events are considered lipid related. The second principle is generality: some biological process is accomplished thorough many mechanisms, which can be are tissue-specific or organism-specific. It is thus important not to conclude too soon that a BP term is lipid related by examining only a single mechanism without considering the others. For example, for BP term GO:0010186 positive regulation of cellular defense response, the only gene products annotated to it are from Arabidopsis thaliana, for which the mechanism contains a key steps involving phospholipid binding, but it cannot be generalized that other organism also have the same key steps since each organism have their own way of regulating cellular defense response.

# References

1. Tan, H. T.; Tan, S.; Lin, Q.; Lim, T. K.; Hew, C. L.; Chung, M. C., Quantitative and temporal proteome analysis of butyrate-treated colorectal cancer cells. *Mol Cell Proteomics* **2008,** 7, (6), 1174-85.

2. Lee, Y. H.; Chung, M. C.; Lin, Q.; Boelsterli, U. A., Troglitazone-induced hepatic mitochondrial proteome expression dynamics in heterozygous Sod2(+/-) mice: two-stage oxidative injury. *Toxicol Appl Pharmacol* **2008,** 231, (1), 43-51.

3. Koenig, T.; Menze, B. H.; Kirchner, M.; Monigatti, F.; Parker, K. C.; Patterson, T.; Steen, J. J.; Hamprecht, F. A.; Steen, H., Robust prediction of the MASCOT score for an improved quality assessment in mass spectrometric proteomics. *J Proteome Res* **2008,** 7, (9), 3708-17.

4. Shilov, I. V.; Seymour, S. L.; Patel, A. A.; Loboda, A.; Tang, W. H.; Keating, S. P.; Hunter, C. L.; Nuwaysir, L. M.; Schaeffer, D. A., The Paragon Algorithm, a next generation search engine that uses sequence temperature values and feature probabilities to identify peptides from tandem mass spectra. *Mol Cell Proteomics* **2007,** 6, (9), 1638-55.

5. Perkins, D. N.; Pappin, D. J.; Creasy, D. M.; Cottrell, J. S., Probability-based protein identification by searching sequence databases using mass spectrometry data. *Electrophoresis* **1999,** 20, (18), 3551-67.

6. Goh, W. W.; Lee, Y. H.; Zubaidah, R. M.; Jin, J.; Dong, D.; Lin, Q.; Chung, M. C.; Wong, L., Network-Based Pipeline for Analyzing MS Data: An Application toward Liver Cancer. *J Proteome Res* **2011,** 10, 2261-72.
