# Supplementary figures and images for: Enhancing the utility of Proteomics Signature Profiling (PSP) with Pathway Derived Subnets (PDSs), performance analysis and specialised ontologies
Source: BMC Genomics. 2013 Jan 16;14:35. doi: 10.1186/1471-2164-14-35 (PMC3636053; doi:10.1186/1471-2164-14-35)

Distribution of counts in mod

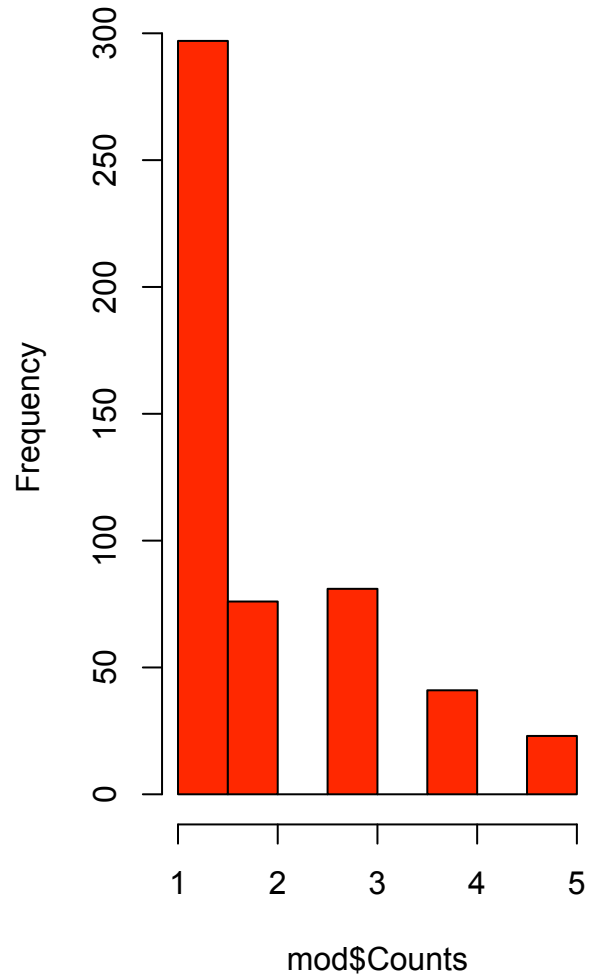

Distribution of counts in poor

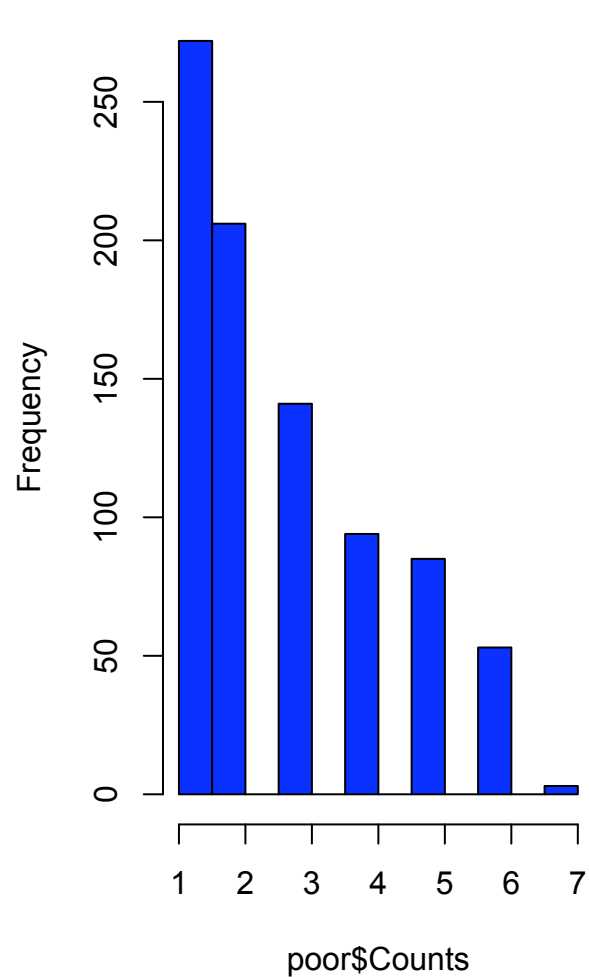

Cluster Dendrogram

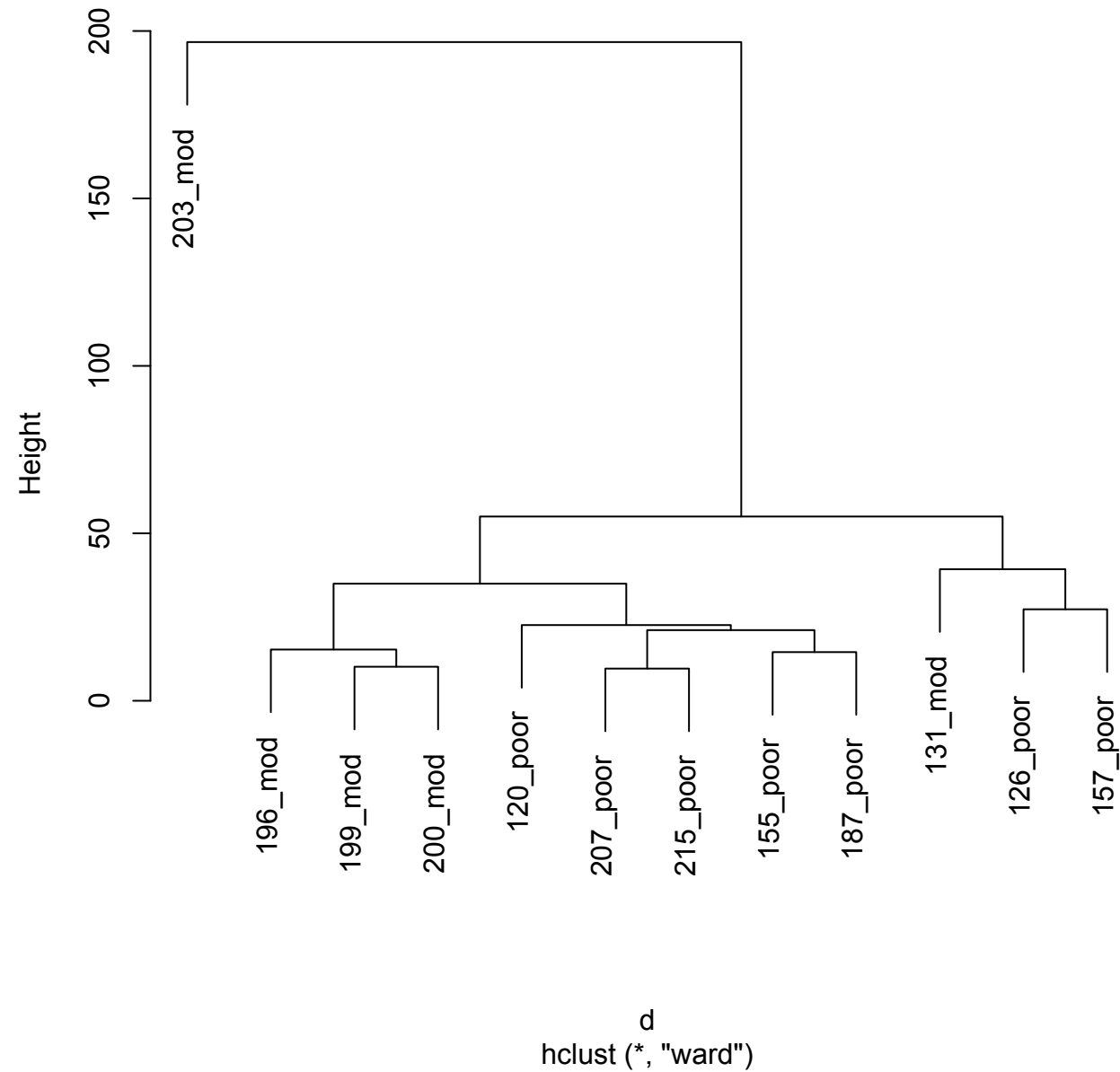

Supplement: Additional file 2: Table S1 — The contributions of each pathway to a PDS. Pathway_ID correspond to the PathwayAPI pathway ID while PDS_contribution indicates the number of PDSs that particular pathway contribute based on the liver cancer proteomics dataset. [file 1471-2164-14-35-S2.pdf]

## size distribution of pathway subnets

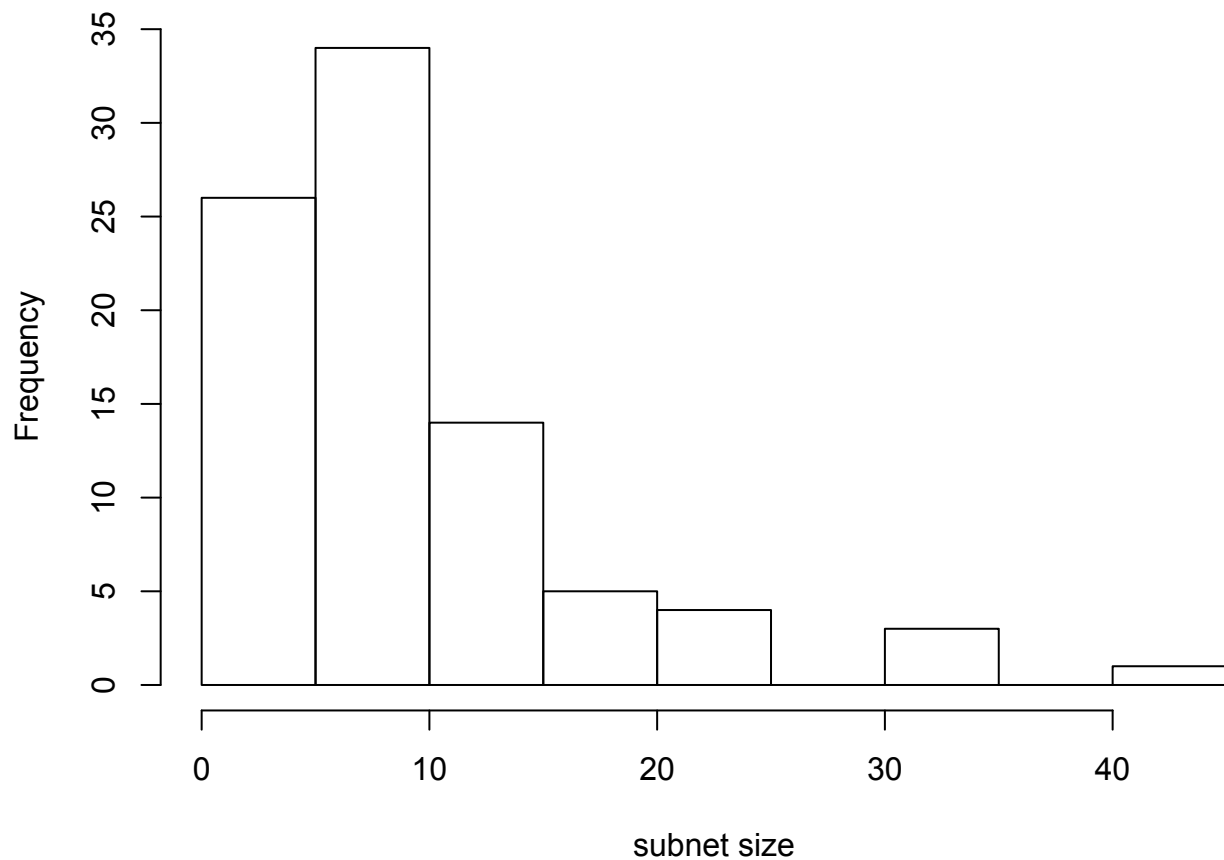

Supplement: Additional file 3: Figure S2 — Size distribution of pathway subnets or PDSs extracted from non-merged PathwayAPI using liver cancer proteomics data. Most PDSs are relatively small, and range from size 5 to 10. [file 1471-2164-14-35-S3.pdf]

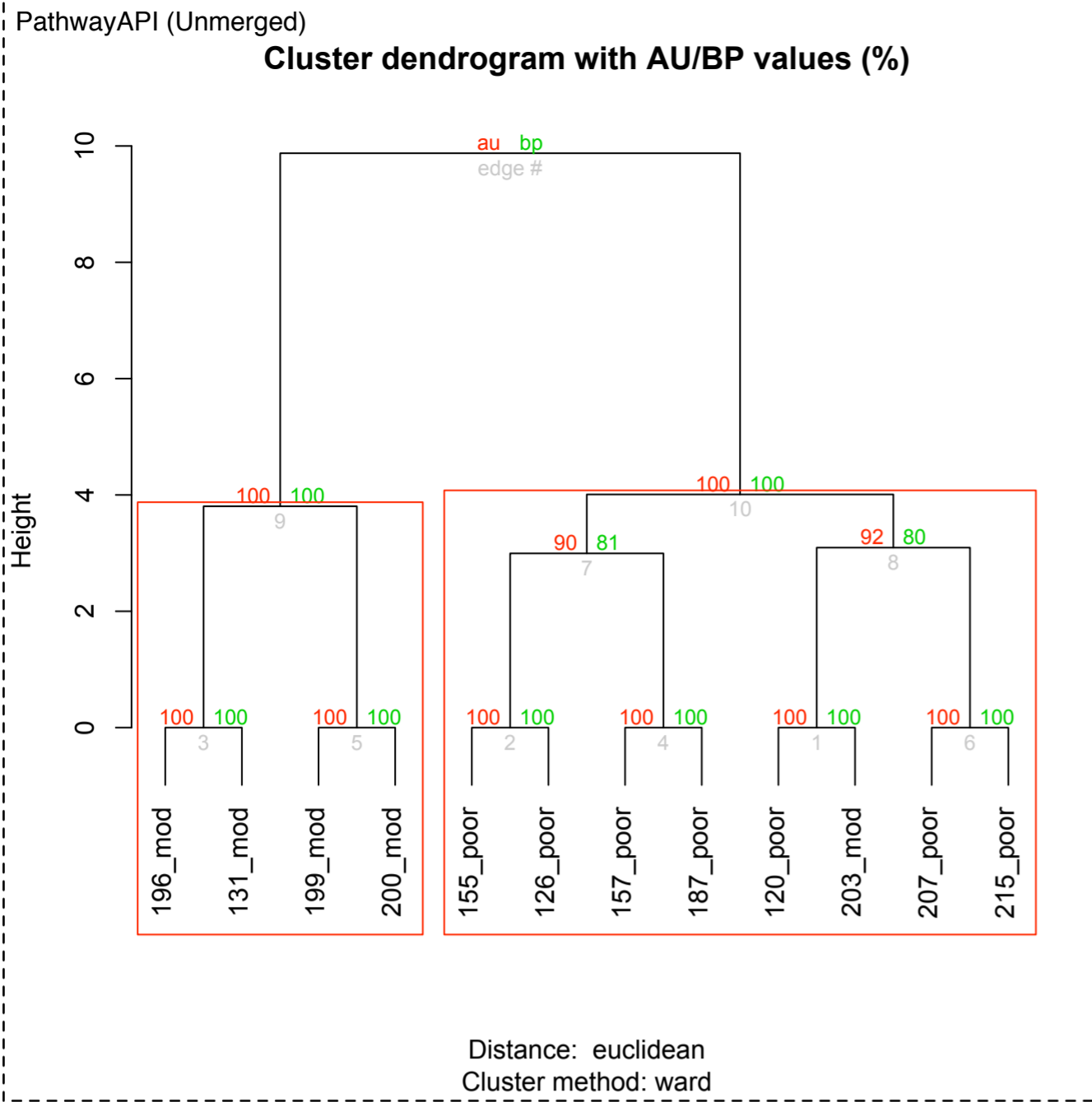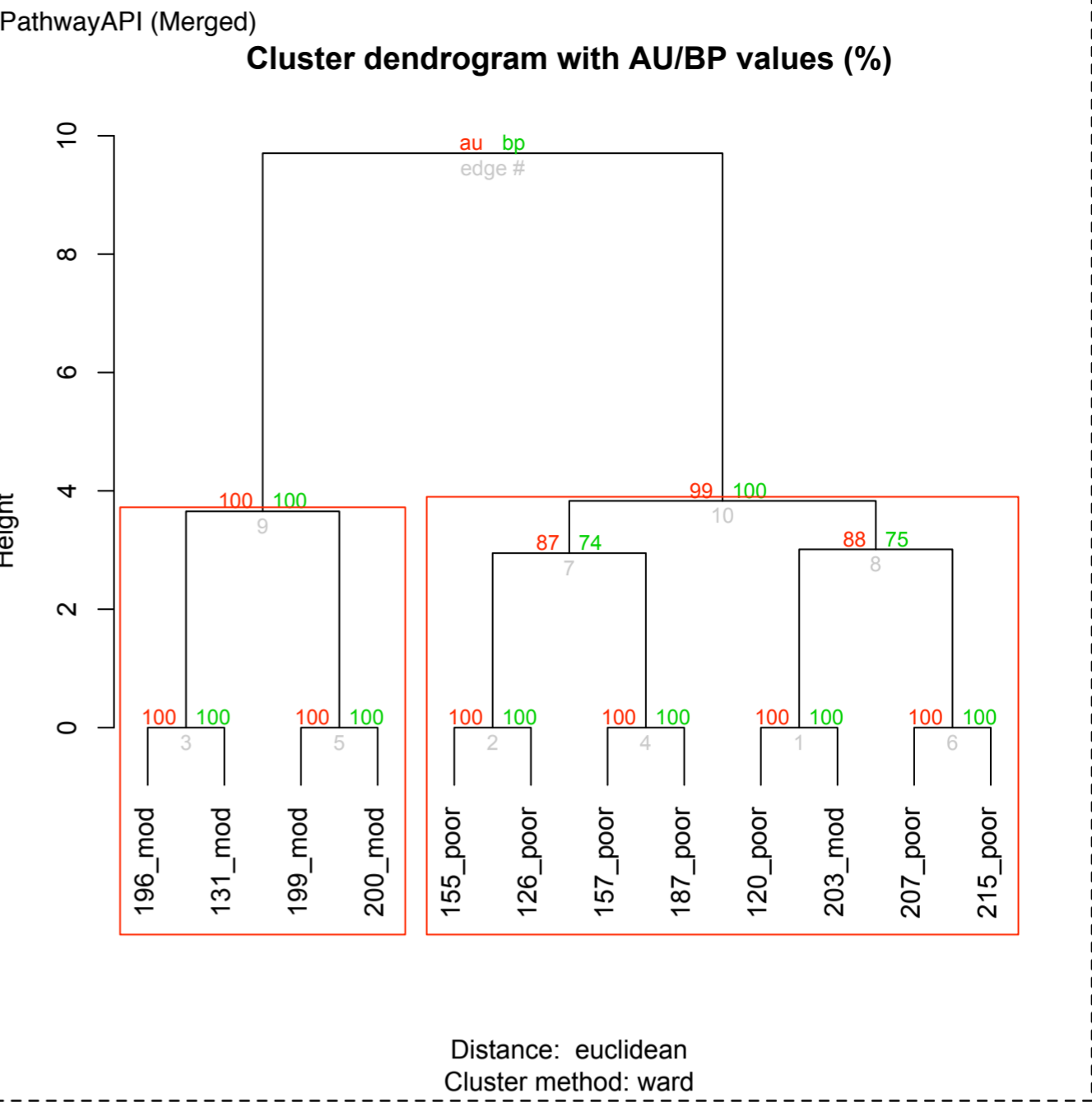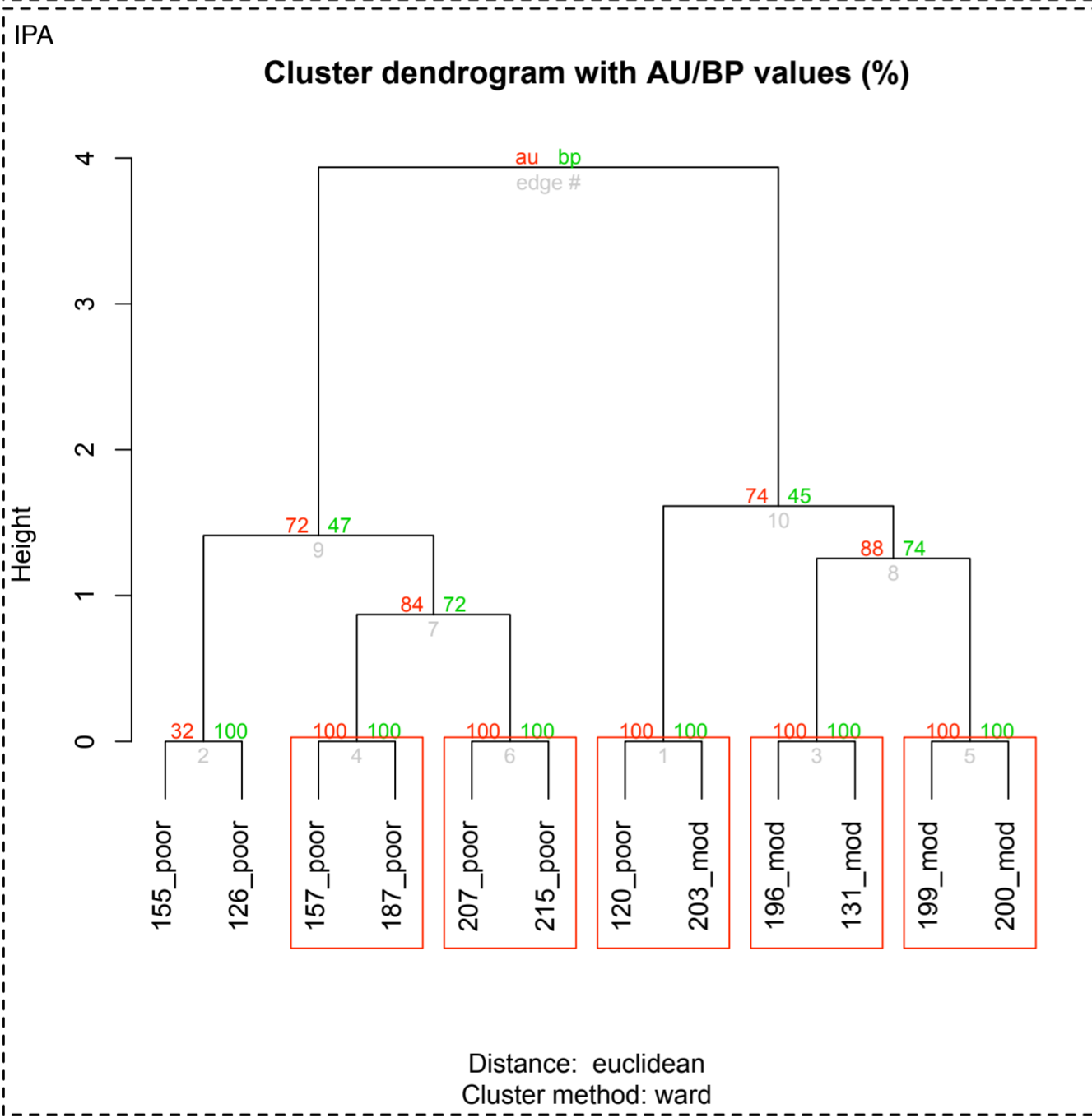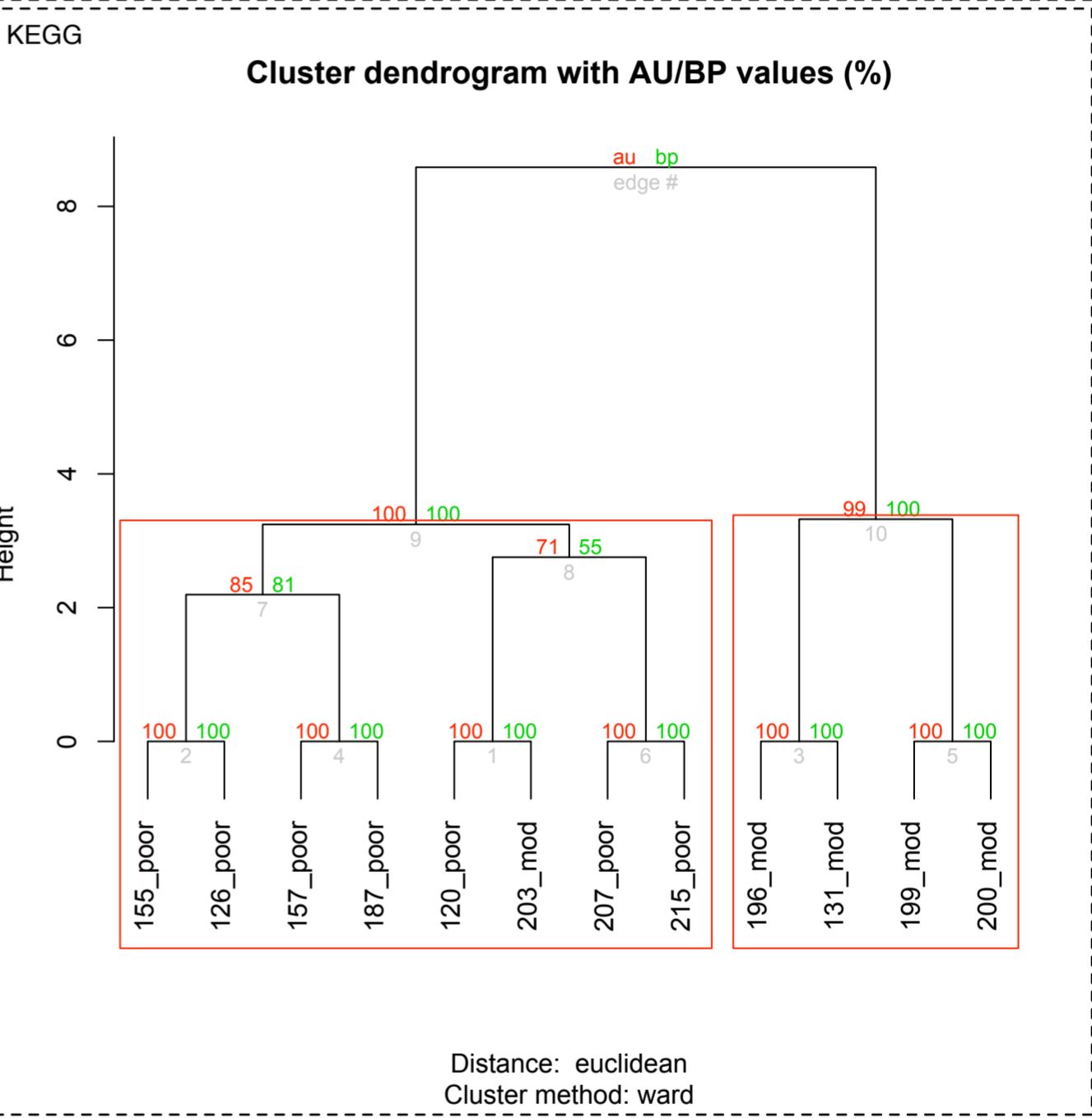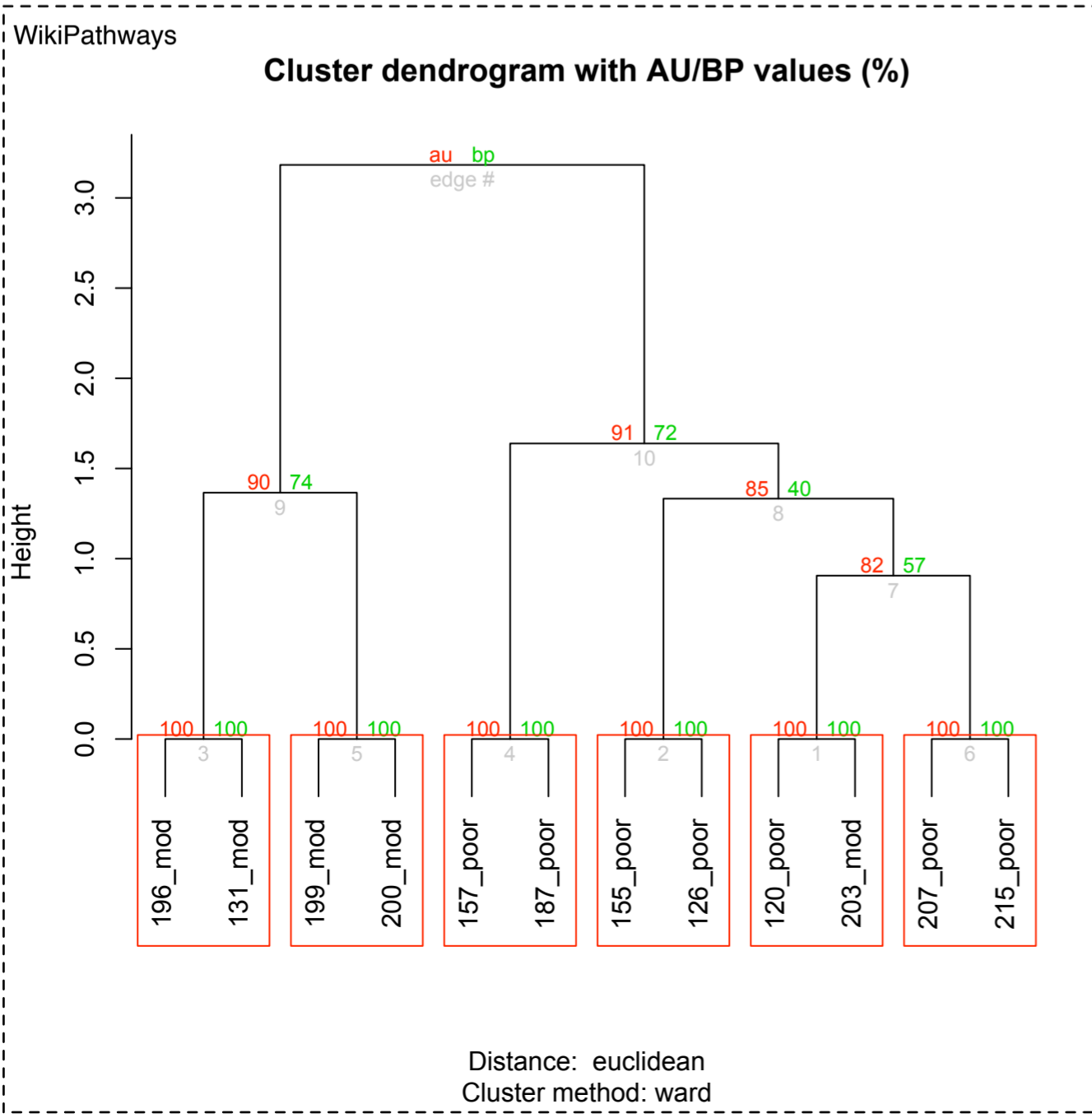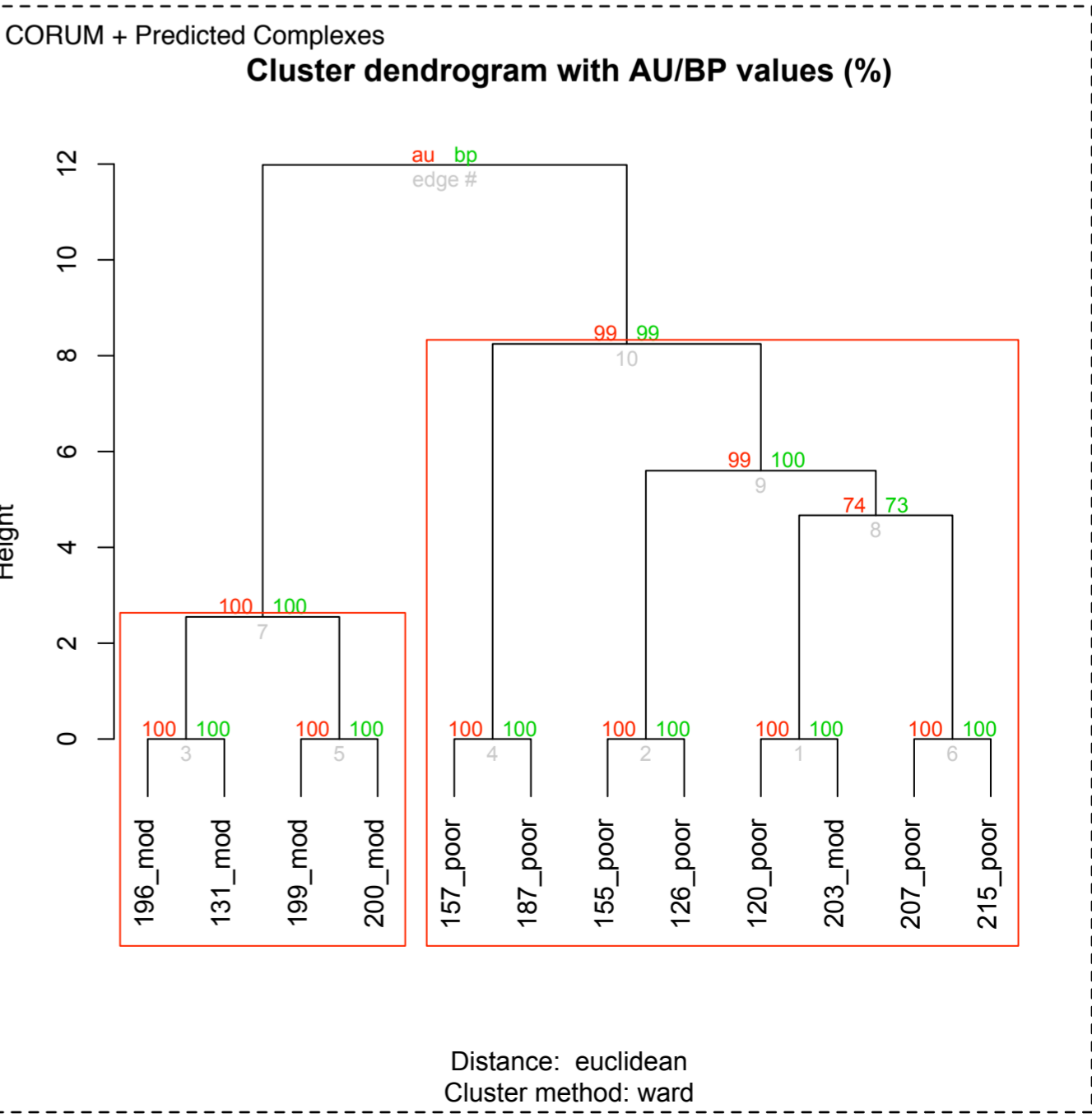

Supplement: Additional file 4: Table S3 — GO term distributions among significant PDSs. [file 1471-2164-14-35-S4.pdf]
